# Promises and Pitfalls of NMDA Receptor Antagonists in Treating Violent Aggression

**DOI:** 10.3389/fnbeh.2022.938044

**Published:** 2022-06-21

**Authors:** Caitlyn J. Bartsch, Jacob C. Nordman

**Affiliations:** ^1^Department of Physiology, University of Southern Illinois Carbondale, Carbondale, IL, United States; ^2^Department of Physiology, University of Southern Illinois School of Medicine, Carbondale, IL, United States

**Keywords:** aggression, early life stress, NMDA receptor, medial amygdala, synaptic plasticity, ketamine, memantine

## Abstract

Treatment options for chronically aggressive individuals remain limited despite recent medical advances. Traditional pharmacological agents used to treat aggression, such as atypical antipsychotics, have limited efficacy and are often replete with dangerous side effects. The non-competitive NMDAR antagonists ketamine and memantine are promising alternatives, but their effects appear to be highly dependent on dosage, context, and personal experience. Importantly, these drugs can increase aggression when combined with substances of abuse or during periods of heightened stress. This is likely due to mechanistic differences operating at specific synapses under different contexts. Previous findings from our lab and others have shown that early life stress, substance abuse, and attack experience promote aggression through NMDAR-dependent synaptic plasticity within aggression-related brain circuits. Ketamine and memantine affect these types of aggression in opposite ways. This has led us to propose that ketamine and memantine oppositely affect aggression brought on by early life stress, substance abuse, or attack experience through opposite effects on NMDAR-dependent synaptic plasticity. This would account for the persistent effects of these drugs on aggression and suggest they could be leveraged as a more long-lasting treatment option. However, a more thorough examination of the effects of ketamine and memantine on cellular and synaptic function will be necessary for responsible administration. Additionally, because the effects of ketamine and memantine are highly dependent on prior drug use, traumatic stress, or a history of aggressive behavior, we propose a more thorough medical evaluation and psychiatric assessment will be necessary to avoid possible adverse interactions with these drugs.

## Introduction

Recurring violent aggression is a major societal concern with few effective treatment options. Currently, the standard of treatment for aggression is antipsychotics, benzodiazepines, or a combination of the two. Unfortunately, these drugs induce numerous side effects, including loss of appetite, fatigue, sleep disturbances, nausea, vomiting, diarrhea, weight gain, increased risk of respiratory depression, and oxygen desaturation (Delbello et al., 2006; Haas et al., 2009; Redden et al., 2009; Geller et al., 2012; Pandina et al., 2012; Pringsheim et al., 2015; Amerio et al., 2018; Pisano et al., 2019; Solmi et al., 2020). In addition, many patients will need to take these drugs for life, increasing their dosage with time as tolerance develops. As a result, current research is devoted to finding a more effective, quick-acting, long-lasting, and well-tolerated alternative.

N-methyl-d-aspartate receptor (NMDAR) antagonists are a promising pharmacological alternative for treating aggressive behavior and aggression-related disorders. NMDARs are members of the ionotropic glutamate binding receptor family (Willard and Koochekpour, 2013). They are composed of five non-identical subunits (GluN1, GluN2A-D, and GluN3A-B) that form a central pore through which cations are conducted upon the binding of glutamate. NMDARs are unique among the ionotropic glutamate binding receptors because of their voltage-gated properties, coincidence detection, and role in synaptic plasticity (Squire and Kandel, 2009; Willard and Koochekpour, 2013). Antagonists for these receptors have been shown to interfere with these properties.

We propose in this review that NMDAR antagonists are favorable in treating aggression over traditional methods due to their quicker onset of action, fewer observed side effects, and potential as a long-lasting treatment option due to their effects on synaptic plasticity (Roberts and Geeting, 2001; Cummings et al., 2008; Wilcock et al., 2008; Hopper et al., 2015; Cole et al., 2016; Riddell et al., 2017; Tran and Mierzwinski-Urban, 2019; Barbic et al., 2021; Nordman et al., 2022). Though there are multiple clinically available NMDAR antagonists, such as the non-competitive antagonists dextromethorphan and amantadine (Hewitt, 2000) and the non-selective, NMDAR-binding opioids methadone, dextropropoxyphene, and ketobemidone (Sang, 2000), we will focus on the non-competitive antagonists ketamine and memantine, as they are the most widely used and successful NMDAR antagonists on the market.

## Ketamine and Memantine in Treating Human Aggression

Ketamine is a non-competitive NMDAR antagonist that has been successfully used to treat aggression in humans. Ketamine was first developed in the United States in the 1960s as an alternative anesthetic to phencyclidine (PCP; Domino et al., 1965). It was found to have a quicker onset of action (less than 5 min) and induce fewer negative emergence symptoms than PCP (Dillon et al., 2003; Tran and Mierzwinski-Urban, 2019), though both produce psychotic-like symptoms in schizophrenia patients (Lahti et al., 2001; Beck et al., 2020). Ketamine gained popularity as a party drug in the 1980s, with sub-anesthetic doses sending users into the colloquially named “K-hole”—a dissociative state commonly accompanied by out-of-body experiences, a sensation of weightlessness, and distortions of time (Dillon et al., 2003). The FDA has approved ketamine for the induction and maintenance of general anesthesia, but there are also many off-label uses of ketamine, including as a local anesthetic, in procedural sedation, pain management, asthma, and depression (Papolos et al., 2013; Burger et al., 2016; Dwyer et al., 2017; Cullen et al., 2018; Tran and Mierzwinski-Urban, 2019; Zarrinnegar et al., 2019; Barbic et al., 2021; Kim et al., 2021; Solano et al., 2021).

Many studies support the use of ketamine to treat violent aggression, agitation, psychosis, self-harm, and suicidal ideation (Burger et al., 2016; Dwyer et al., 2017; Cullen et al., 2018; Zarrinnegar et al., 2019). In the majority of these cases, clinicians take advantage of ketamine’s sedative properties to control aggressive outbursts in hospital and pre-hospital settings (Roberts and Geeting, 2001; Melamed et al., 2007; Le Cong et al., 2012; Scheppke et al., 2014; Riddell et al., 2017; Barbic et al., 2021; Kent et al., 2022). Sedative doses of ketamine fall in the 1–2 mg/kg range if given intravenously, and 3–5 mg/kg if given intramuscularly. These doses are necessary to sedate acutely aggressive individuals for the safety of the patient and staff but are also associated with higher rates of intubation, emergence delirium, and other adverse effects (Woods and Almvik, 2002; Cole et al., 2016; Chang et al., 2019). Because of these risks, a low-dose treatment protocol should be followed when possible.

Sub-sedative doses of ketamine (0.2–0.5 mg/kg IV, 30–120 mg intranasal) have been successful in treating aggression in psychiatric patients (Papolos et al., 2013; Burger et al., 2016; Dwyer et al., 2017; Zarrinnegar et al., 2019). For instance, intranasal ketamine significantly reduced aggression in children and adolescents with pediatric bipolar disorder (Papolos et al., 2013). Additionally, military patients presenting to the hospital with depression and suicidal ideations experienced a rapid improvement of symptoms within 40 min of administration of ketamine (Burger et al., 2016). Numerous studies support ketamine’s rapid-acting antidepressant properties when given at low doses (Papolos et al., 2013; Burger et al., 2016; Dwyer et al., 2017; Zarrinnegar et al., 2019). Interestingly, chronic ketamine use has been found to be more addictive at these sub-sedative doses (Morgan et al., 2004; Bonnet, 2015; Schak et al., 2016). Therefore, ketamine treatment for aggression symptoms related to psychiatric disease must be carried out in a regulated, controlled fashion. These studies are discussed in the *Synaptic Plasticity* section below. A summary of our review findings on ketamine in aggression is outlined in Table 1.

**Table 1:** Human studies on the effects of ketamine and memantine.

**References**	**Observation**	**Comments**
**Ketamine**
Roberts and Geeting (2001)	A single dose of 5 mg/kg IM ketamine suppressed violent behavior within 2–3 min through sedation	Lorazepam was administered immediately afterward
Cole et al. (2016)	A single dose of 5 mg/kg IM ketamine sedated individuals with severe acute undifferentiated agitation significantly faster than 10 mg IM haloperidol bolus.The mean time to adequate sedation was 5 min vs. 17 min, respectively.	Ketamine resulted in a higher rate of intubation and other complications
Barbic et al. (2021)	A single dose of 5 mg/kg IM ketamine outperformed 5 mg IM midazolam and 5 mg IM haloperidol boluses in suppressing severe psychomotor agitation in hospital patients through sedation	
Riddell et al. (2017)	Mean single dose of 2.97 mg/kg IM and 0.87 mg/kg IV ketamine outperformed 5.71 mg/kg IM haloperidol, 2.25 mg/kg IM and 3.08 mg/kg IV midazolam, and 2.40 mg/kg IM and 1.90 mg/kg IV lorazepam in treating severely agitated emergency department patients through sedation	Dosing was not uniform throughout the study, average doses are listed.
Burger et al. (2016)	A single dose of 0.2 mg/kg IV ketamine decreased depression and suicidal ideation seen in military members that met the criteria for inpatient psychiatric admission	
Dwyer et al. (2017)	0.5 mg/kg IV ketamine infused 7x over an 8-week hospitalization decreased depressive symptoms and suicidal ideation in an adolescent with severe treatment-resistant depression. Outpatient treatments every 3–6 weeks after discharge with continued improvement in symptoms	
Papolos et al. (2013)	30–120 mg intranasal ketamine every 3–7 days elicited a substantial reduction in measures of mania, fear of harm, and aggression in youth with pediatric bipolar disorder- fear of harm phenotype	
Solano et al. (2021)	A single dose of 400 mg IM ketamine bolus used to treat excited delirium with concurrent cocaine intoxication had a statistically significant increased rate of intubation in ED	Symptoms of excited delirium include aggressive behavior, combativeness, and agitation
Zarrinnegar et al. (2019)	0.5 mg/kg IV ketamine treatment 6x over a 3-week inpatient hospitalization ameliorated symptoms of psychosis, self-harm, and suicidality in an adolescent with severe treatment-resistant depression. Suppression of aggressive symptoms persisted for several months following discharge.	Prior treatment with many antidepressants, benzodiazepines, and antipsychotics failed to improve symptomology
Scheppke et al. (2014)	A single dose of 4 mg/kg ketamine IM suppressed aggression through sedation in 96% of patients	Average time of 2 min to achieve suitable sedation
Le Cong et al. (2012)	Acute treatment of 0.5–1.5 mg/kg IV ketamine suppressed aggressive outbursts through sedation	Ketamine treatment was initiated when benzodiazepines and/or antipsychotics failed
Melamed et al. (2007)	Acute treatment of IV ketamine (dosage/concentration not listed) through sedation decreased agitation in soldiers suffering a traumatic injury	Was effective alone or in combination with other sedative agents
Donoghue et al. (2015)	Acute treatment with 10 mg procedural IV ketamine-induced an 8 and 13-day remission of aggressive behaviors in a child with PTSD after a tonsillectomy and sedated MRI.	
Mankowitz et al. (2018)	A mean, single dose of 315 mg IM ketamine effectively treated undifferentiated agitation through sedation	
Heydari et al. (2018)	A single dose of 4 mg/kg IM ketamine outperformed 5 mg IM haloperidol bolus to sedate severely agitated patients in the emergency department	
O’Connor et al. (2019)	A single dose of 4 mg/kg IM ketamine had a higher intubation rate than haloperidol (5 mg IM bolus) plus benzodiazepines (2–4 mg IM bolus) in severely agitated patients	
Olives et al. (2016)	A single dose of 5 mg/kg IM ketamine delivered pre-hospital was found to be associated with a high rate of intubation (63%) in profoundly agitated patients	
Kent et al. (2022)	A single dose of 5 mg/kg IM ketamine outperformed the combination of haloperidol (5 mg IM bolus) and midazolam (5 mg IM bolus) to sedate severely agitated ED patients through sedation. The mean time to adequate sedation was 5.8 min vs. 14.7 min, respectively	The ketamine arm experienced a higher rate of serious adverse events
**Memantine**		
Cummings et al. (2008)	20 mg/day PO memantine treatment agitation/aggression in patients with Alzheimer’s disease and baseline levels of agitation/aggression	
Wilcock et al. (2008)	20 mg/day PO memantine decreased agitation and aggression in patients with Alzheimer’s disease	
Thomas and Grossberg (2009)	20 mg/day PO memantine treatment was associated with less severity or emergence of agitation/aggression compared to placebo	
Da Re et al. (2015)	20 mg/day PO memantine used to treat dementia resulted in improvement of agitation in 19% of participants, but an increase in agitation in 5.6% of participants	
Herrmann et al. (2011)	Significant decrease in agitation and aggression following 10 mg PO memantine treatment twice daily in patients with Alzheimer’s disease	
Gauthier et al. (2008)	20 mg/day PO memantine reduced agitation and aggression in patients with Alzheimer’s disease compared to the placebo group	
Ichinose et al. (2021)	20 mg/day PO memantine successfully treated aggressive behavior that was a byproduct of hepatic encephalopathy	
Kishi et al. (2017)	10 and 20 mg/day PO memantine treatment significantly improved agitation/aggression when compared to the control group	
Fox et al. (2012)	10 mg PO memantine twice daily did not significantly improve agitation in patients with moderate to severe Alzheimer’s disease	

*Abbreviations: IM, intramuscular; IV, intravenous; PO, per os/by mouth; ED, emergency department*.

Memantine is a non-competitive NMDAR antagonist, with low to moderate affinity, which is approved for treating moderate to severe Alzheimer’s disease and dementia (Huey et al., 2005; Robinson and Keating, 2006; Thomas and Grossberg, 2009). It has been reported that 5%–10% of Alzheimer’s patients and 96% of dementia patients exhibit aggressive behavior over the course of their illness (Keene et al., 1999; Jaclson and Mallory, 2009). Atypical antipsychotics are among the most common treatments for agitation and aggression in Alzheimer’s and dementia patients (Ballard et al., 2006, 2008; Cerejeira et al., 2012), but there is a significant risk of mortality, stroke, hallucination, and relapse after continued use, especially during the first 30 days of treatment (Kales et al., 2012; Calsolaro et al., 2019).

A key benefit of memantine is that, due to its long biological half-life, it is not used recreationally, is not habit-forming, and is well tolerated. Notably, memantine at a dose of 10–20 mg/day has been effective in reducing aggression and agitation associated with Alzheimer’s disease and dementia patients without the accompanying risks of atypical antipsychotics (Cummings et al., 2008; Wilcock et al., 2008). However, some patients experience treatment-induced agitation with memantine use (Da Re et al., 2015), suggesting that a more careful clinical examination is key to proper treatment. A summary of our review findings on memantine in aggression is outlined in Table 1.

The future of ketamine and memantine is promising. Still, our limited knowledge of the mechanisms behind NMDARs and aggression, paired with the diverging effects observed in animal models warrants more research, which we discuss below.

## NMDARs, Ketamine, and Memantine in Animal Aggression

**Figure 1:**
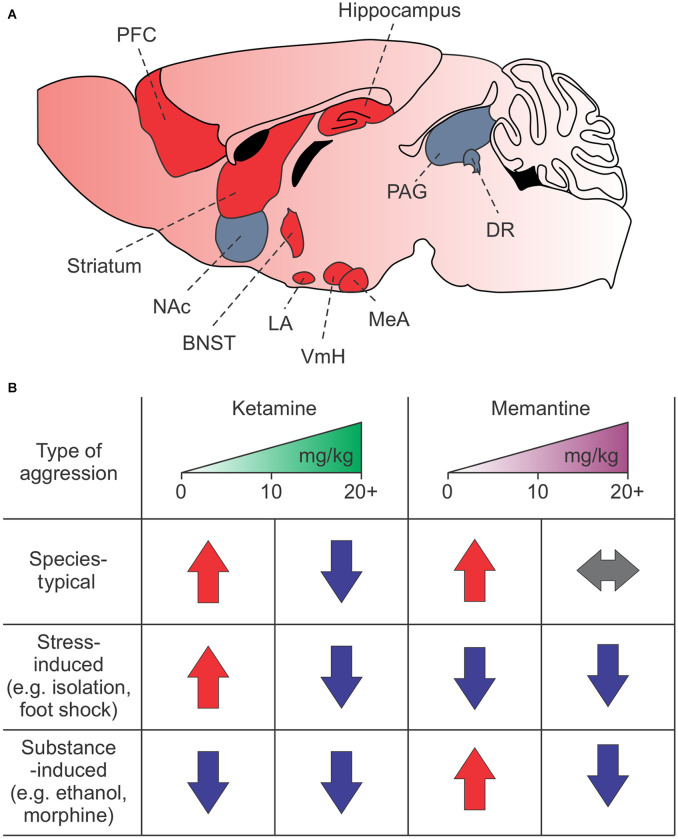
Summary of NMDAR-dependent aggression in mice. **(A)** Brain regions associated with attack behavior are enriched in and regulated by NMDARs. Red regions indicate known areas involved in NMDAR-dependent synaptic plasticity-induced aggression. **(B)** Effects of low or high doses of ketamine and memantine on species-typical, stress-induced, and drug or alcohol-induced aggression. Red arrows—increased aggression; blue arrows—decreased aggression; gray arrows—no change in aggression. The PFC, prefrontal cortex; NAc, nucleus accumbens; BNST, bed nucleus of the stria terminalis; LA, lateral amygdala; VmH, ventromedial hypothalamus; MeA, medial amygdala; PAG, periacqeductal gray; DR, dorsal raphe.

NMDARs are highly expressed in regions associated with attack behavior, such as the amygdala, prefrontal cortex (PFC), hippocampus, nucleus accumbens, hypothalamus, striatum, and brain stem (Shaikh and Siegel, 1994; Shaikh et al., 1994; Petralia et al., 2007; Peregud et al., 2012; Takahashi et al., 2015; Bacq et al., 2018; Chen and Hong, 2018; Newman et al., 2018; Zoicas and Kornhuber, 2019; Falkner et al., 2020; Figure 1A). The NMDARs, particularly the GluN2 subunits, have a nuanced and varied role in species-typical and excessive aggression, as demonstrated by rodent studies. For example, decreased GluN2B expression in the lateral amygdala is associated with naturally occurring and social isolation-induced aggression (Bacq et al., 2018). However, increased GluN2A and GluN2B expression and GluN2B-dependent NMDAR currents in the hippocampus and frontal cortex are associated with social isolation-induced aggression and morphine-induced aggression, respectively (Meyer et al., 2004; Zhao et al., 2009; Chang et al., 2015; Chang and Gean, 2019). Interestingly, in the mPFC, increases in GluN2D, but not GluN2A or GluN2B, may mediate alcohol-induced aggression (Newman et al., 2018).

Unsurprisingly, ketamine and memantine also have a nuanced role in aggression that depends on dosage, context, experience, and species (Table 2). For example, in mice and zebrafish, high doses of ketamine suppress territorial aggression while low doses increase it (Newman et al., 2012; Michelotti et al., 2018). In rats, low doses, but not high doses, of ketamine suppress muricide behavior (Takahashi et al., 1984). Memantine, by contrast, has no effect on species-typical aggression (Sukhotina and Bespalov, 2000; Newman et al., 2012, 2018).

**Table 2:** Animal studies on the effects of ketamine and memantine.

**References**	**Sex/Age/Species**	**Observation**
**Ketamine**		
Nordman et al. (2022)	7–8-week-old male mice	IP injections of 10 mg/kg ketamine enhance early life stress-induced aggression
Newman et al. (2018)	Adult male mice	IP injections of 3–10 mg/kg ketamine increased aggression IP injections of 30 ketamine mg/kg decreased aggression IP injections of 30 mg/kg ketamine decreased alcohol-heightened and alcohol non-heightened aggression
Newman et al. (2012)	Adult male mice	IP injections of 3–10 mg/kg ketamine increased aggression IP injections of 30 mg/kg decreased aggression IP injections of 30 mg/kg ketamine decreased alcohol-heightened and alcohol non-heightened aggression
Michelotti et al. (2018)	Adult zebrafish, 50:50 male:female	Low dose (2 mg/kg) ketamine increased aggression High (20–40 mg/kg) dose reduced aggression
Takahashi et al. (1984)	Male rats	IP injections of low doses (3–5 mg/kg) of ketamine increased aggression in rats deprived of REM sleep and in mice after social isolation Mouse killing behavior response blocked in muricide rats at all ketamine doses administered IP
Covington et al. (2018)	Adult male rats and mice	IP injections of low dose (10 mg/kg) ketamine reduced motivated aggression IP injection of low doses (7.5 and 10 mg/kg) of ketamine reduced alcohol-escalated motivated aggression
Shin et al. (2019)	Adolescent mice	Chronic (7 days) IP injections of 15 mg/kg ketamine reduced aggression induced by neonatal maternal separation
**Memantine**		
Nordman et al. (2022)	7–8-week-old male mice	IP injections of MK-801 and memantine suppress early life stress-induced aggression
Newman et al. (2018)	Adult male mice	IP injection of 1–10 mg/kg memantine increased aggression 20–30 ug/ul memantine infused into the mPFC increased aggression in mice that consumed alcohol
Newman et al. (2012)	Adult male mice	IP injection of 1–10 mg/kg memantine increased aggression in mice that consumed ethanol IP injection of 17 mg/kg memantine decreased aggression compared to vehicle controls
Sukhotina and Bespalov (2000)	Adult male mice	IP injection of 10–30 mg/kg memantine lessened morphine withdrawal-facilitated aggression

*Abbreviations: Intraperitoneal, IP*.

As with humans, substance use plays a significant role in the effects of ketamine and memantine on aggression. In alcohol-challenged mice, ketamine reduces territorial aggression at high doses and reduces motivated aggression at low doses (Newman et al., 2012; Covington et al., 2018). Memantine increases alcohol-heightened aggression at low doses but has no effect at high doses (Newman et al., 2012, 2018). By contrast, high doses of memantine, but not low doses, decrease morphine withdrawal-facilitated aggressive behavior in mice (Sukhotina and Bespalov, 2000).

Stress also appears to be a strong factor. For example, ketamine increases aggression in sleep-deprived rats and socially isolated mice (Takahashi et al., 1984). In adolescent mice, ketamine decreases aggression induced by neonatal maternal separation (Shin et al., 2019) but increases aggression induced by chronic social isolation followed by acute non-contingent foot shock (Nordman et al., 2022). Interestingly, memantine suppresses aggression induced by chronic social isolation stress followed by acute non-contingent foot shock, similar to what has been seen with the non-competitive NMDAR antagonist MK-801 (Chang et al., 2015; Nordman et al., 2022).

While these findings clearly demonstrate a role for NMDARs in aggression and NMDAR antagonists as a potential treatment option, dosage, context, experience, and brain region all need to be considered (Figure 1B). Furthermore, the effects of ketamine and memantine suggest they operate through distinct mechanisms, which we discuss in the “*Mechanistic Differences*” section below.

## Synaptic Plasticity

NMDARs are one of the primary sources of synaptic plasticity in the brain (Squire and Kandel, 2009; Willard and Koochekpour, 2013). When bound by glycine and glutamate at certain voltage thresholds, NMDARs conduct large amounts of the second messenger cation calcium into the synapse. This sudden, large increase in calcium concentration activates a series of kinases and phosphatases that promote the insertion or removal of another type of ionotropic glutamate binding receptor, the AMPA receptor. AMPA receptors are not voltage-gated, so the more AMPA receptors there are in the synapse, the more the membrane will be depolarized when AMPARs are bound by glutamate, thus increasing the likelihood that a neuron will fire an action potential. Conversely, the fewer AMPA receptors there are in the synapse, the less the membrane will be depolarized when AMPARs are bound by glutamate, thus decreasing the likelihood that a neuron will fire an action potential when activated. Insertion and removal of AMPA receptors by NMDARs is the principal mechanism for synaptic plasticity at excitatory synapses in the brain.

Excessive chronic aggression brought on by early life stress and social isolation is mediated by the persistent effects of NMDAR-dependent synaptic plasticity, which can be suppressed using NMDAR antagonists (Chang et al., 2015, 2018; Nordman et al., 2020a, b). Therefore, NMDAR antagonists possess a feature more popular mood-stabilizing and aggression-suppressing drugs lack: they can inhibit certain persistent forms of maladaptive social behaviors such as excessive and recurring aggression from ever forming, likely through their effects on synaptic plasticity. This is best seen in animal models, where a single dose can inhibit depression-like, anxiety-like, or aggressive behavior when administered before a potentiating event (da Silva et al., 2010; Ma et al., 2013; Yang et al., 2016; Chang et al., 2018; Nordman et al., 2020a, b) or reverse maladaptive behaviors when administered after the potentiating event (Maeng et al., 2008; Moda-Sava et al., 2019).

In a recent study, we showed that NMDAR antagonism could alter aggression induced by early life stress after a single dose (Nordman et al., 2022). Our early life stress paradigm involves social isolation during early adolescence followed by acute physical stress in the form of non-contingent foot shock during late adolescence. Combining these stressors produces prolonged increases in excessive aggression when measured seven days later. When we systemically injected a single dose of the non-competitive NMDAR antagonists MK-801 or memantine immediately before foot shock in our early life-stress paradigm, no increase in aggression was found when measured 7 days later. However, when we administered a single intraperitoneal (IP) injection of ketamine before foot shock, surprisingly, we observed a significant increase in aggression when measured 7 days later. These results indicate that different NMDAR antagonists have distinct effects on early life stress-induced aggression and suggest that memantine and ketamine are mechanistically distinct, possibly through their opposing effects on synaptic plasticity.

In support of this, in another recent study, we showed that NMDARs regulate aggression priming through NMDAR-dependent synaptic plasticity (Nordman et al., 2020a). Attack priming is a phenomenon where previous attack experience increases the likelihood and severity of a future attack within a narrow time window (~30–60 min). Notably, in that study, we found that attack experience, high-frequency optogenetic stimulation of aggression brain pathways, and early life stress heightened aggression by synaptically potentiating glutamatergic synapses within the aggression circuit. Specifically, *in vivo* electrophysiological recordings of optically evoked excitatory postsynaptic potentials indicate that both attack experience and acute footshock after social isolation potentiate glutamatergic synapses between the posterior ventral segment of the medial amygdala (MeApv) and its downstream synaptic partners the ventrolateral aspect of the ventromedial hypothalamus (MeApv-VmHvl) and the medial aspect of the bed nucleus of the stria terminalis (MeApv-BNSTm; Nordman et al., 2020a, b). A single systemic injection of the non-competitive NMDAR antagonist MK-801 30 min before aggression testing suppressed both aggression priming and synaptic potentiation at these synapses, indicating that both are NMDAR-dependent.

Since (1) aggression priming and early life stress-induced aggression both involve synaptic plasticity within the same glutamatergic MeApv pathways; and (2) aggression priming is NMDAR-dependent, it stands to reason that early life stress-induced aggression is NMDAR-dependent as well. However, the distinct effects of MK-801, memantine, and ketamine on aggression suggest these drugs have opposing effects on synaptic plasticity within MeApv pathways. Future studies should explore this possibility.

Nevertheless, NMDAR antagonists are an exciting treatment option for excessive aggression and aggression associated with early life stress because it bypasses one of the great challenges of traditional pharmacological methods: daily and lifelong administration of a dangerous drug that only diminishes in its efficacy over time. In support of this, it has been found that in some psychiatric patients, ketamine can suppress aggressive behavior long past the time it takes for the body to metabolize the drug (plasma half-life is 79 +/– 8 min; Hirota and Lambert, 1996; Donoghue et al., 2015; Dwyer et al., 2017; Zarrinnegar et al., 2019). For example, a single sedating dose (10 mg) of ketamine successfully suppressed aggressive behavior for 13 days in a 7-year-old child diagnosed with PTSD displaying reactive attachment disorder and disruptive behavior disorder (Donoghue et al., 2015). Three months later, the same sedating dose of ketamine reduced aggression and increased the patient’s receptivity to psychiatric care for another 8 days, showing that ketamine can be effectively readministered while retaining its persistent effects on aggression. Similarly, patients receiving ketamine treatment for depression showed significant improvements in aggression symptoms (e.g., psychosis, self-harm, and suicidality) that lasted for weeks to months afterward (Dwyer et al., 2017; Zarrinnegar et al., 2019). Synaptic plasticity could explain the vast difference between the time it takes to metabolize ketamine and the sustained decrease in aggressive symptoms seen in these patients.

Therefore, in this review, we argue that NMDAR antagonists like ketamine and memantine could reduce the frequency of taking, or even alleviate the patient’s need to remain on, increasingly tolerant and dangerous drugs like antipsychotics, benzodiazepines, lithium, or anticonvulsants for the treatment of aggression. In addition, we argue that NMDAR antagonists could be used to suppress aggression induced by a potentiating event like stress. Of course, it is difficult to predict when a potentiating event might occur. However, NMDAR antagonists could suppress the susceptibility of individuals with psychiatric diseases to develop aggressive behavior during periods of heightened stress or substance abuse, a significant advantage over current options. We note though that the distinct effects of ketamine and memantine on aggression associated with early life stress in our animal model suggest the need for great care when prescribing NMDAR drugs to treat excessive aggression.

## Mechanistic Differences

We highlight ketamine and memantine in this review as they are the most common and successful NMDAR-drugs for treating excessive aggression in humans and are potent synaptic plasticity blockers. While both drugs are similar in their effects on channel function (Johnson et al., 2015), important differences remain. Perhaps the most important differences are the receptor types each drug targets.

For example, ketamine is non-selective for the NMDAR, binding to muscarinic, monoaminergic, and opioid receptors, among others (Hirota and Lambert, 1996). It has been hypothesized that these interactions mediate the psychotomimetic effects many patients experience and may account for the more persistent effects of ketamine on pain and as an antidepressant (Sleigh et al., 2014; Zorumski et al., 2016). This would suggest that the effects of ketamine on aggression-related disorders are not exclusively mediated through NMDARs.

Ketamine and memantine also differ in their NMDAR dissociation rates, with ketamine binding to the NMDAR for longer periods of time than memantine (Johnson et al., 2015; Glasgow et al., 2017). This has been used to explain ketamine’s high and memantine’s low sedative and psychotomimetic effects (Lanthorn et al., 2000; Bolshakov et al., 2003; Kotermanski and Johnson, 2009; Kitanaka et al., 2018).

The location of NMDARs may also explain the differences between these drugs. There is evidence that memantine binds more readily to extrasynaptic NMDARs than ketamine (Zhao et al., 2006; Leveille et al., 2008; Okamoto et al., 2009; Milnerwood et al., 2010; Johnson et al., 2015), though these results have been disputed (Wroge et al., 2012; Emnett et al., 2013; Zhou et al., 2013). Extrasynaptic NMDARs have been associated with mood and anxiety disorders related to chronic social defeat stress (Tse et al., 2019). Ketamine, however, likely does not distinguish between synaptic and extrasynaptic NMDARs (Autry et al., 2011; Emnett et al., 2013; Nosyreva et al., 2013; Gideons et al., 2014; Miller et al., 2014).

It is also possible that memantine and ketamine bind to overlapping but distinct NMDAR subpopulations. For example, it was recently shown that memantine and ketamine target different NMDAR subtypes, with memantine preferentially binding to and desensitizing the GluN1/2A subtype and ketamine binding to and desensitizing the GluN1/2B subtype (Glasgow et al., 2017).

Another possibility is that the enhanced effects of ketamine are due to the disinhibition of excitatory signaling in different areas of the brain. Ketamine has been shown to disinhibit pyramidal neurons in the medial prefrontal cortex (mPFC) and the CA1 region of the hippocampus by inhibiting GABAergic interneurons in both areas (Moghaddam et al., 1997; Widman and McMahon, 2018; Ali et al., 2020; Gerhard et al., 2020). This is hypothesized to occur because of ketamine’s higher affinity for GluN2B-containing NMDARs expressed in PFC somatostatin interneurons (Ali et al., 2020; Gerhard et al., 2020) and GluN2D-containing NMDARs preferentially expressed in CA1 hippocampal interneurons (Perszyk et al., 2016; Zanos and Gould, 2018). It is interesting to consider that a similar mechanism may be operating at MeApv-VmHvl and MeApv-BNSTm synapses, enhancing synaptic potentiation that drives early life stress-induced aggression.

Finally, it is worth noting that ketamine is a chiral compound, with most ketamine available today being a racemic mixture of the two optical enantiomers (R) and (S) (Kohrs and Durieux, 1998; Paul et al., 2009; Hashimoto, 2019). (R)-ketamine and (S)-ketamine have differing effects in many human and rodent studies (Paul et al., 2009; Hashimoto, 2019; Rafalo-Ulinska et al., 2022). It was initially suggested that (S)-ketamine was a better candidate as an antidepressant than (R)-ketamine because of its higher affinity for the NMDAR (Singh et al., 2016). And in fact, the FDA recently approved (S)-ketamine in the form of a nasal spray for treatment-resistant depression in adults (Hashimoto, 2019). However, in recent studies, (R)-ketamine was found to have greater potency and fewer side effects at lower doses than (S)-ketamine, suggesting it might be a safer alternative (Chang et al., 2019; Hashimoto, 2019; Beck et al., 2020; Rafalo-Ulinska et al., 2022). In a meta-analysis of 36 studies, racemic and (S)-ketamine were associated with a statistically significant increase in transient psychopathology in healthy and schizophrenia patients (Beck et al., 2020). Prescribing the lowest dose of ketamine necessary is essential due to the high risk of abuse associated with ketamine. Ketamine users quickly develop tolerance to the drug, and typical withdrawal effects include cravings, anxiety, sweating, and shaking (Kokane et al., 2020).

These differences in synaptic location, NMDAR subtype, chirality, and tolerance may explain why memantine and ketamine have such diverging clinical and experimental effects and provide a mechanism for the unique effects of these drugs on excessive and stress-induced aggression.

## Clinical Assessment

Our studies and others have demonstrated that different NMDAR antagonists can promote or exacerbate aggressive behavior depending on a prior history of substance abuse, traumatic stress, or aggressive behavior. A proper assessment would decrease the incidence of aggression by ruling out those NMDAR antagonists as treatment options. Therefore, a complete evaluation of the patient’s history of psychiatric and physical illness and an assessment of past substance use should be performed before administering and prescribing these drugs. Fortunately, there are standard methods for assessing whether an individual has a history of traumatic stress or aggressive behavior or is currently using or abusing drugs or alcohol that would exclude them from being prescribed specific NMDAR antagonists.

Tests that evaluate a history of traumatic stress include The Primary Care PTSD Screen for DSM-5 (Prins et al., 2016), The Short Post-Traumatic Stress Disorder Rating Interview (Connor and Davidson, 2001), and the Trauma Screening Questionnaire (Brewin et al., 2002). Tests for individuals with a history of aggressive behavior or who are in an actively aggressive state include the Dynamic Appraisal of Situational Aggression (DASA; Ogloff and Daffern, 2006) and the Brøset Violence Checklist (BVC; Woods and Almvik, 2002), which assess the likelihood that a patient will become aggressive through factors such as physical or verbal threats, negative attitudes, and impulsivity. Finally, tests like the Tobacco, Alcohol, Prescription Medication, and Other Substance Use (TAPS) Tool (McNeely et al., 2016) and the Brief Screener for Alcohol, Tobacco, and Other Drugs (BSTAD) tool (Kelly et al., 2014) are used to evaluate drug and alcohol abuse in adults and adolescents, respectively. Importantly, these assessment tools can be implemented in the hospital setting, significantly reducing the likelihood of unintended aggressive behavior by NMDAR antagonists, and paving the way for more nuanced administration.

## Conclusion

This review highlights some of the promises and pitfalls of the non-competitive NMDAR antagonists, and in particular ketamine and memantine, in treating excessive and recurring violent aggression. On the one hand, NMDAR antagonists are clearly powerful clinical tools in managing violent aggression. They possess a quicker onset of action and fewer observed side effects than current alternatives. NMDARs also have great potential as a long-lasting treatment option due to their effects on synaptic plasticity (Roberts and Geeting, 2001; Cummings et al., 2008; Hopper et al., 2015; Cole et al., 2016; Riddell et al., 2017; Barbic et al., 2021), where these drugs can induce persistent changes in synaptic function, neural firing, and animal behavior, in some cases, even after a single dose (Wilcock et al., 2008; Moda-Sava et al., 2019; Tran and Mierzwinski-Urban, 2019; Nordman et al., 2022).

While the potential of these drugs to treat excessive aggression related to changes in synaptic plasticity is enormous, care is needed. Numerous reports show that NMDAR antagonists have highly varied effects on aggression depending on dose, context, and experience (Nordman, 2021), outlined in Figure 1B. While many of these drugs can lessen or even suppress aggression, they can also heighten aggression when used improperly (Sukhotina and Bespalov, 2000; Newman et al., 2012, 2018; Covington et al., 2018; Nordman et al., 2022). Evidence from our lab and others suggests these differences may depend on changes in synaptic plasticity within limbic circuits (Figure 1A). Future studies should be aimed at investigating this intriguing possibility.

## Data Availability Statement

The original contributions presented in the study are included in the article, further inquiries can be directed to the corresponding author.

## Author Contributions

CB and JN wrote the manuscript. All authors contributed to the article and approved the submitted version.

## Conflict of Interest

The authors declare that the research was conducted in the absence of any commercial or financial relationships that could be construed as a potential conflict of interest.

## Publisher’s Note

All claims expressed in this article are solely those of the authors and do not necessarily represent those of their affiliated organizations, or those of the publisher, the editors and the reviewers. Any product that may be evaluated in this article, or claim that may be made by its manufacturer, is not guaranteed or endorsed by the publisher.
